# The Role of Perioperative Endoscopic Retrograde Cholangiopancreatography and Biliary Drainage in Large Liver Hydatid Cysts

**DOI:** 10.1155/2014/301891

**Published:** 2014-11-09

**Authors:** A. Krasniqi, B. Bicaj, D. Limani, M. Maxhuni, A. Rrusta, F. Hoxha, A. Hamza, V. Zejnullahu, F. Sada, S. Hashani, R. Musa, R. Latifi

**Affiliations:** ^1^Faculty of Medicine, University of Prishtina, 10000 Prishtina, Kosovo; ^2^Department of Surgery, University Clinical Centre of Kosova, 10000 Prishtina, Kosovo; ^3^Department of Surgery, The University of Arizona, Tucson, AZ 87524, USA

## Abstract

*Background*. The best surgical technique for large liver hydatid cysts (LHCs) has not yet been agreed on. *Objectives*. The objective of this study was to examine the role of perioperative endoscopic retrograde cholangiopancreatography (ERCP) and biliary drainage in patients with large LHCs. *Methods*. A 20-year retrospective study of patients with LHCs treated surgically at the University Clinical Center of Kosovo (UCCK). We divided patients into 2 groups based on treatment period: 1981–1990 (Group I) and 2001–2010 (Group II). Demographic characteristics (sex, age), the surgical procedure performed, complications rate, and outcomes were compared. *Results*. Of the 340 patients in our study, 218 (64.1%) were female with median age of 37 years (range, 17 to 81 years). 71% of patients underwent endocystectomy with partial pericystectomy and omentoplication, 8% total pericystectomy, 18% endocystectomy with capitonnage, and 3% external drainage. In Group I, 10 patients underwent bile duct exploration and T-tube placement; in Group II, 39 patients underwent bile duct exploration and T-tube placement. In addition, 9 patients in Group II underwent perioperative ERCP with papillotomy. The complication rate was 14.32% versus 6.37%, respectively (*P* = 0.001). *Conclusion*. Perioperative ERCP and biliary drainage significantly decreased the complication rate and improved outcomes in patients with large LHCs.

## 1. Introduction

Hydatid disease is a zoonotic infection caused by larval stages of dog tapeworms belonging to the genus* Echinococcus* (family Taeniidae); it is also referred to as echinococcosis [1]. Three broad morphologic forms of echinococcosis are recognized clinically: cystic, alveolar, and polycystic echinococcus.

Human cystic echinococcosis (the most common), caused by* Echinococcus granulosus*, is the most common presentation—probably accounting for more than 95% of the estimated 2 to 3 million cases of hydatid disease worldwide [2]. This disease continues to be a substantial cause of morbidity and mortality in many parts of the world [1]. Hydatidosis is endemic in Mediterranean countries and in other sheep- and cattle-raising regions [3]. In southeastern Europe, despite the lack of exact data on the incidence of human cystic echinococcosis, liver hydatid cysts (LHCs) and lung hydatid cysts continue to be very common on surgical wards [4]. The most frequent anatomic sites for echinococcal cysts are the liver (70% to 80%) and the lungs (15% to 25%); occurrence in other sites is very rare [3–8]. The morphologic classifications of LHCs include the sonographic Gharbi's classification [9, 10], the new World Health Organization classification [11], and systems based on topography and complications [12].

Although the concept of managing liver hydatidosis is changing, surgery combined with scolicidal therapy is still the gold standard for complete cure [13]. Controversy persists regarding the appropriate surgical technique [14]. Open surgical procedures remain the most appropriate choice for large, complicated, and multiple LHCs [4, 13, 15, 16], but opinions and experiences vary regarding the type of procedure: tissue-sparing techniques (endocystectomy with partial pericystectomy) versus radical operations (complete pericystectomy, liver resection) [15, 17–21]. Endocystectomy with partial pericystectomy is the most frequent procedure for the surgical management of complicated, large, and multiple LHCs [4, 17, 18].

Cystobiliary communication increases the perioperative complication rate and necessitates additional tests during surgery in order to identify and properly close biliary leaks [19, 22]. Two recent approaches for patients with large LHCs have not yet been clearly evaluated in the literature: (1) the combination of additional endoscopic procedures (such as ERCP and other drainage procedures of the biliary tree) with standard surgical treatment and (2) minimally invasive procedures [23–26]. However, for patients with complicated LHCs, we noted a trend toward perioperative ERCP with sphincterotomy [15, 27]. Further studies should be encouraged in this field [28, 29].

In this study, our main objective was to compare the outcomes (in particular, the complication rates) of 2 groups of patients with large LHCs who were treated surgically at our institution, in 2 different eras—with a focus on the role of perioperative ERCP and biliary drainage.

## 2. Methods

**Figure 1:**
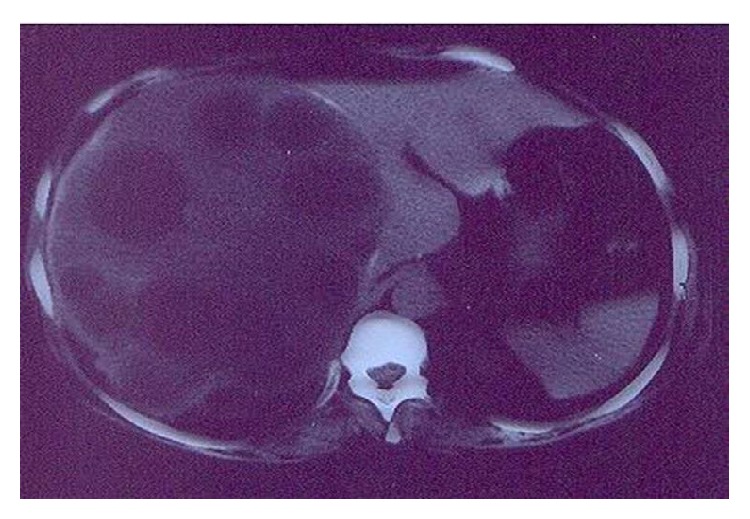
Large liver hydatid cysts (diameter of >12 cm) as per ultrasound (US) findings.

In this 20-year retrospective review, we analyzed the records of patients with large LHCs who were treated surgically at the University Clinical Center of Kosovo (UCCK) of the University of Prishtina, Kosovo. We divided patients into 2 groups according to their treatment period: 1981–1990 (Group I) and 2001–2010 (Group II). Then, we compared demographic characteristics (e.g., sex and age), the surgical procedure performed, outcomes (e.g., resolution of the cyst), postoperative hospital length of stay, and complication rates. We defined large LHCs as those with a diameter, per ultrasound (US), of more than 12 cm (Figure 1). To analyze the data, we used Student's *t*-test (Sigma Stat, version 2.0). We considered a *P* value < 0.05 to be statistically significant. The study has been approved by the institution.

## 3. Results

**Figure 2:**
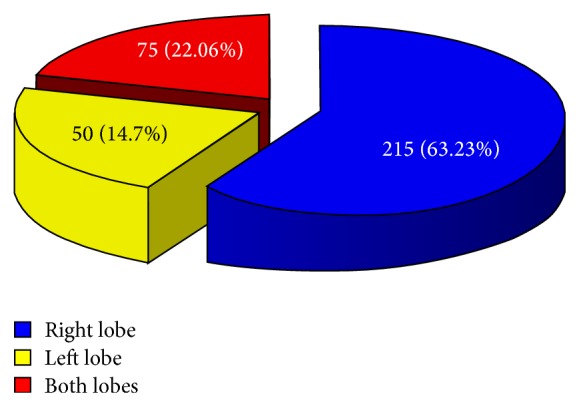
Localization of cysts in the liver.

**Figure 3:**
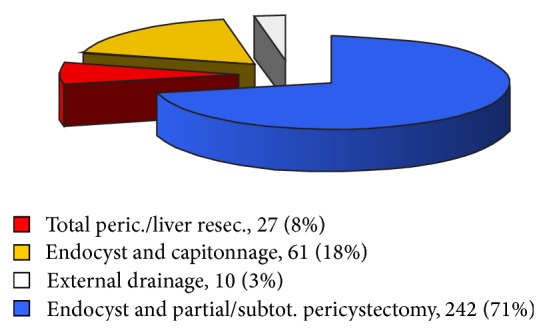
Surgical procedures.

Of the 340 patients in our study group, 218 (64.1%) were female and 122 (35.8%) were male (a ratio of 1.8 : 1). The median age was 37 years (range, 17 to 81 years). Hepatomegaly was the most common clinical presentation (53.6%), followed by an abdominal mass (30.9%), increased temperature (19.2%), and jaundice (10%). Often patients had more than one presenting symptom. In most patients (63.2%, *n* = 215), the cyst was localized in the right lobe; in 22.06% (*n* = 75) it was localized in the left lobe; and in 14.7% (*n* = 50) it was localized in both lobes (Figure 2). Most of the patients (71%; *n* = 242) underwent endocystectomy with partial pericystectomy and omentoplication (Figure 3). The other operations (29%; *n* = 98) included total pericystectomy (8%; *n* = 27), endocystectomy with capitonnage (18%; *n* = 61), and external drainage (3%; *n* = 10). In Group II, capitonnage and external drainage were almost entirely abandoned. In Group I, 10 patients underwent bile duct exploration and T-tube placement; in Group II, 39 patients underwent bile duct exploration and T-tube placement. In Group I, none of the patients underwent perioperative ERCP with papillotomy; in Group II, 9 patients underwent perioperative ERCP with papillotomy.

For our entire study group (*n* = 340), the mean postoperative hospital length of stay was 16.6 days (range, 5 to 71 days): 18.8 days in Group I versus 11.8 days in Group II. The difference between the 2 groups was not significantly different.

**Figure 4:**
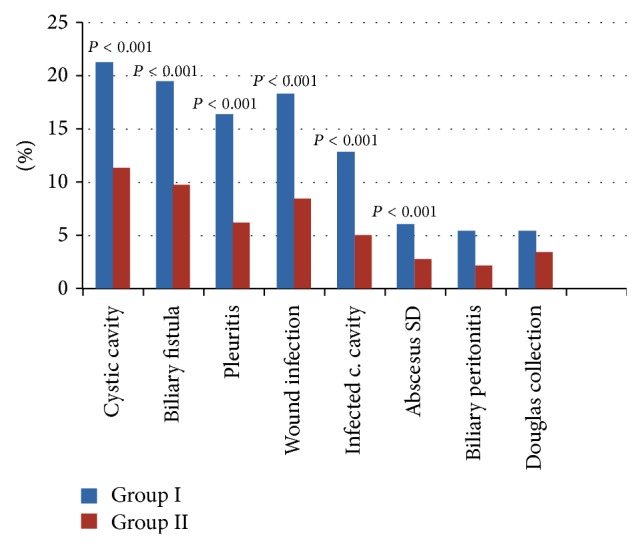
Postoperative complication rate between the two groups.

The overall complication rate was 14.32% in Group I versus 6.37% in Group II (*P* = 0.001). The main postoperative complications were prolonged persistence of a residual cystic cavity, pleuritis, biliary fistulas, wound infections, cystic cavity infections, subdiaphragmatic abscesses, and biliary peritonitis (Table 1). In Group I, 1 patient died because of a complication; in Group II, none died. The overall complication rate between the 2 groups was significantly different (Figure 4).

## 4. Discussion

**Figure 5:**
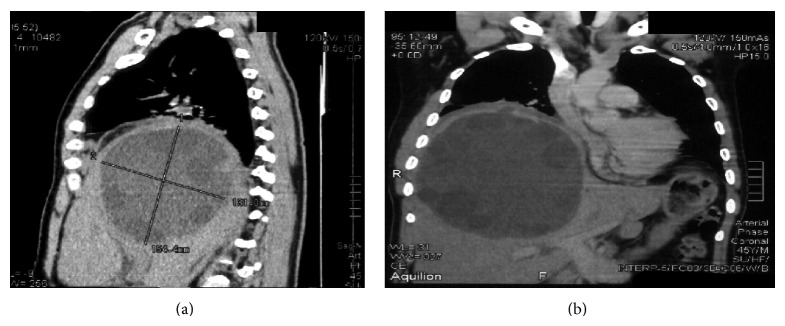
CT scan of large liver hydatid cysts < 12 cm with significant elevation of the right diaphragm.

No prospective randomized clinical study has been done to establish standard guidelines and agreement on the most appropriate surgical technique in patients with large LHCs. Surgeons in endemic areas, which have more complicated cases, usually prefer conservative surgical procedures, because they consider resection procedures too radical and extensive for benign lesions [4, 16–21]. In our earlier studies [4], we used different surgical procedures, but, in 71% of those patients, we applied tissue-sparing techniques (endocystectomy with partial pericystectomy). In large LHCs, particularly those in central anatomic sites (Figures 5(a) and 5(b)), the rate of their communication with large bile ducts is high. In such patients, radical surgical procedures can cause serious postoperative complications. Therefore, we think that patients with large LHCs can be treated safely by conservative surgery. A further consideration is that liver hydatid disease mainly occurs in developing countries with modest hospital equipment and limited resources.

In our current study, we used abdominal US to initially diagnose all of our patients, although most Group II patients underwent CT. We did not employ any preoperative radiologic classification system in Group I patients, but we did in most Group II patients. All cysts were classified according to our intraoperative findings (e.g., unilocular, multilocular, degenerated, suppurated, multiple, and with intrabiliary communication).

**Figure 6:**
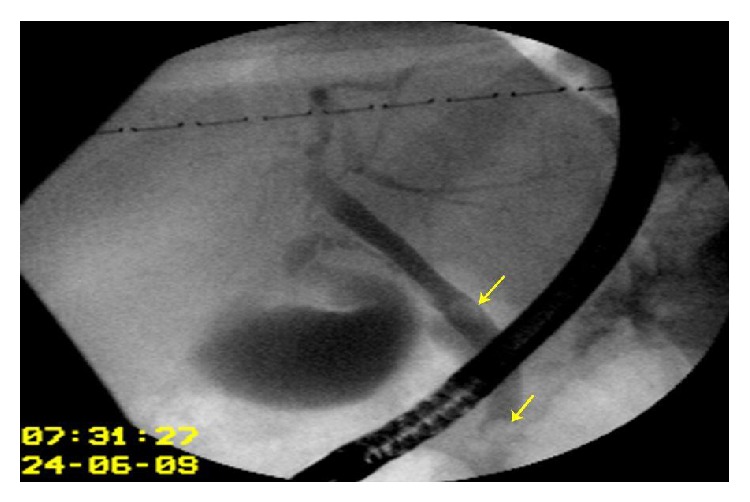
Preoperative ERCP shows compression of the right hepatic duct by the large hydatid cyst and presence of daughter cysts in the main bile duct as well, in a patient with jaundice. Endoscopic papillotomy and daughter cysts extraction were done preoperatively.

**Figure 7:**
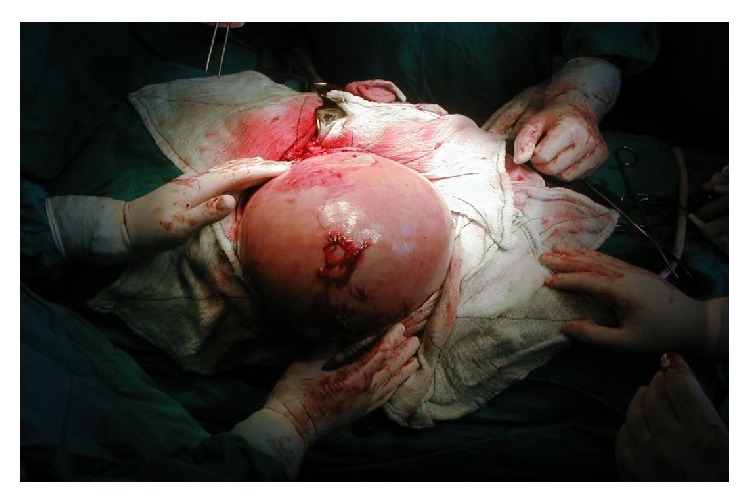
Adhesion dissection freed the cyst from other organs. Intracystic scolicidal injection and prevention of intraperitoneal spillover were done with 25% NaCl solution.

**Figure 8:**
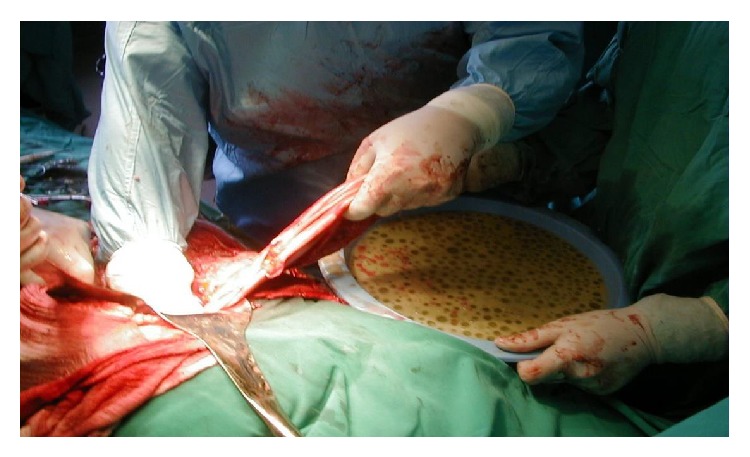
Removal of cystic content and maximal reduction of pericyst.

**Figure 9:**
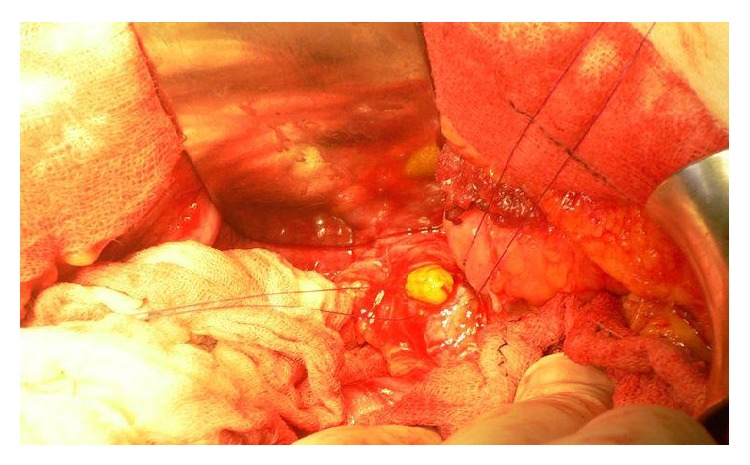
After careful treatment of the remaining cavity and closure of the eroded bile channels, the exploration of the bile duct is needed in cases with cystobiliary fistulas, jaundice, and dilated bile duct. Photo shows the daughter cyst in the main bile duct.

**Figure 10:**
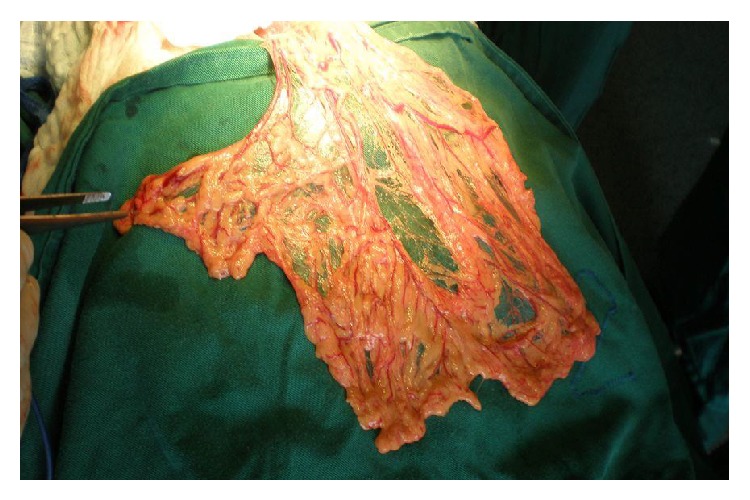
The omental flap prepared for filling the remaining cavity and omentopexy.

**Figure 11:**
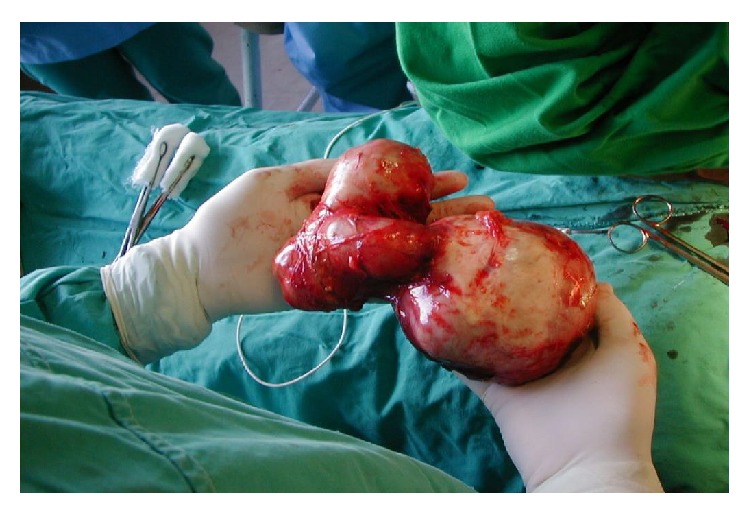
Complete specimen of complex hydatid cyst removed.

Our surgical approach to the liver was through a subcostal incision. Intraoperative steps consisted of cyst identification and evaluation, adhesion dissection, careful prevention of intraperitoneal spillover and intracystic scolicidal therapy (25% NaCl), evacuation of contents and total endocystectomy with partial pericystectomy (in most patients), careful examination of the cystic cavity, blue-ink identification of biliary leaks (and placement of 3.0 polydioxanone (PDS) sutures), bile duct exploration for daughter cysts, T-tube placement in case of erosion of a larger bile channel, omentoplication, and drainage of the cystic cavity (Figures 6, 7, 8, 9, 10, and 11). Our choice of surgical procedure was based on the size of the cyst, its location, the presence of intrabiliary communication, age of the patient, the ability to use ERCP, and our team preference.

In recent decades, the development of new imaging and invasive diagnostic procedures [24, 29] has changed the concept of surgical treatment of patients with liver hydatidosis [13, 22, 23]. Although many new treatment procedures are now in use, the postoperative complication rate still remains very high [19, 23, 30, 31]. The most frequent serious postoperative complications are prolonged persistence of a residual cystic cavity, cystic cavity infections, biliary peritonitis, and biliary fistulas. A number of studies have described the use of additional endoscopic and other interventional procedures in an attempt to reduce cystic cavity persistence and infections, biliary leaks, biliary fistulas, and other complications [19, 22, 30, 31]. Kayaalp et al. [19] showed that intraoperative identification of the portion of the biliary tree involved with the cyst and the suturing of any cystobiliary communication significantly reduced postoperative complications after hydatid liver surgery. Adas et al. [31] concluded that ERCP and related therapeutic procedures were safe and valuable in the postoperative management of external biliary fistulas in patients with hepatic hydatid disease, while Akbulut et al. [32] reported that ERCP and related therapeutic procedures in the early postoperative period decreased the development of biliary peritonitis and the requirement for reoperative intervention. Other authors [33–35] have stressed the importance of careful closure of biliary fistulas inside the residual cavity, omentopexy, and obliteration of the cavity itself.

At our institution in recent years, we used ERCP with endoscopic sphincterotomy as an additional perioperative diagnostic and therapeutic procedure in 9 patients (preoperatively in 3 and postoperatively in 6). In all 9 of those patients, that particular procedure very effectively helped us pinpoint the cystobiliary communication, bile duct obstruction, and the best way to extract retained daughter cysts—thereby helping avoid development of jaundice and bile accumulation in the residual cystic cavity. We were able to intraoperatively identify, with blue ink, high-output biliary fistulas and then carefully suture them. In all, 10 patients in Group I and 39 in Group II underwent bile duct exploration and T-tube placement.

The main indications for preoperative ERCP were complex cysts associated with jaundice, which had diagnostic, but more importantly therapeutic, role. By decompressing the biliary system in a timely fashion, we believe that we prevented major complications of obstructive jaundice. On the other hand, postoperative ERCP has its role particularly in cases where intraoperatively multiple cysts are found. Using ERCP, one will potentially identify new biliary tree-cysts communications and readily decompress biliary tree. In both cases, we believe that ERCP has significant role and will improve overall patient's condition.

In comparing the outcomes in our 2 groups, we found that decompression of the biliary tree (via ERCP with endoscopic sphincterotomy and T-tube drainage of the bile duct) significantly decreased the complication rate and hospital length of stay (Figure 4) (*P* = 0.001). When we obliterated the residual cystic cavity and sutured inside the cavity of bile fistulas combined with via ERCP with endoscopic sphincterotomy and T-tube drainage of the bile duct, we observed positive effects, namely, prevention of bile collection and of suppuration into the residual cavity, as well as reduction of the persistence of a residual cavity and reduction of the number of biliary fistulas. Clearly, ERCP with sphincterotomy and T-tube drainage of the bile duct provided good results in terms of decreasing the postoperative complication rate in patients with large LHCs.

In conclusion, we found that the combination of ERCP and biliary drainage (in conjunction with sphincterotomy, bile duct exploration, and T-tube placement) significantly decreased the complication rate and improved outcomes in patients with large LHCs. Our postoperative complication rates were significantly lower in Group II. We recommend perioperative additional diagnostic ERCP and, eventually, endoscopic sphincterotomy in such patients.

## Figures and Tables

**Table 1:** Postoperative complication rate.

	Cystic cavity^*^	Biliary fistula^*^	Pleuritis^*^	Wound infection^*^	Infected cystic cavity^*^	Subdiaphragmatic abscess^*^	Biliary peritonitis^*^	Douglas collection^*^
Group I	(35) 21.34	(32) 19.51	(27) 16.46	(30) 18.29	(21) 12.8	(10) 6.09	(9) 5.48	(9) 5.48
Group II	(20) 11.36	(17) 9.65	(11) 6.25	(15) 8.52	(9) 5.11	(5) 2.84	(4) 2.27	(6) 3.4

^*^Values in parentheses are percentages.

## Abbreviations

LHC: Liver hydatid cyst
ERCP: Endoscopic retrograde cholangiopancreatography
US: Ultrasound
CT: Computed tomography.
